# Short term exposure to titanium, aluminum and vanadium (Ti 6Al 4V) alloy powder drastically affects behavior and antioxidant metabolites in vital organs of male albino mice

**DOI:** 10.1016/j.toxrep.2018.06.006

**Published:** 2018-06-13

**Authors:** Ghulam Khadija, Ayisha Saleem, Zafrin Akhtar, Zahra Naqvi, Maham Gull, Mahnoor Masood, Sana Mukhtar, Momna Batool, Nida Saleem, Tahir Rasheed, Naira Nizam, Ather Ibrahim, Furhan Iqbal

**Affiliations:** a Zoology Division, Institute of Pure and Applied Biology, Bahauddin Zakariya University, Multan, 60800, Pakistan; b Institute of Advanced Materials, Bahauddin Zakariya University, Multan, 60800, Pakistan

**Keywords:** Titanium aluminium vanadium Alloy, Albino mice, Behavior, Hematology, Antioxidants

## Abstract

Titanium, Aluminum and Vanadium (Ti 6Al 4V) alloy are frequently used as surgical implant but regarding their compatibility in living systems is limited. Ti 6Al 4V was prepared from high purity constituents and Ti 6Al 4V alloy powder (25 mg/ml solvent/Kg body weight) was gavaged to albino mice for 8 days. A saline treated control group was maintained in parallel. A series of behavioral (rota rod, light and dark box, open field and novel object) test performance, complete blood count, selected serum (HDL cholesterol, LDL cholesterol, creatinine, cholesterol and triglycerides) parameters, antioxidant metabolites from vital organs (superoxide dismutase, catalase and lipid peroxidation) from vital organs and body weight were determined in both treatments. It was observed that rota rod test performance in male (P = 0.05) and novel object recognition capability in female mice (P = 0.04) were significantly reduced as compared to their respective control groups. Body weight, complete blood count and studied serum parameters remained unaffected when compared between two treatments of both genders. Concentration of superoxide dismutase in liver (P = 0.008), heart (P = 0.01) and lungs (P = 0.05) was significantly elevated while catalase concentration in liver (P = 0.001) was significantly decreased in female albino mice that were exposed to 25 mg/ml solvent/kg body weight of Ti 6 A l 4 V alloy powder. In case of male albino mice, superoxide dismutase concentration in lungs was reduced (P = 0.05) in mice exposed to Ti 6 A l 4 V alloy powder.

In conclusion, our results indicated that short term exposure to 25 mg/ml solvent/Kg body weight of Ti-6Al-4V alloy powder supplementation had adversely affected selected aspects of behavior of albino mice in a gender specific manner. Analysis of antioxidant parameters in vital organs has demonstrated that the applied dose of Ti-6Al-4V alloy powder can disturb the H₂O₂ associated metabolic pathways in albino mice, especially in female mice. As this alloy is part of surgical implants, so we recommend that their effects in living systems must be extensively explored under variable dose and exposure time conditions to know more about their biocompatibility.

## Highlights

• Body weight, complete blood count and studied serum parameters remained unaffected upon Ti 6 A l 4 V alloy powder exposure.
• Short tern exposure to Ti 6Al 4V powder drastically affected neuromuscular coordination in male mice during rota rod test.
• Reduced novel object recognition ability in female mice exposed to Ti 6Al 4V alloy powder.
• Disturbed antioxidant metabolites in vital organs of mice treated with Ti 6Al 4V alloy powder.

## Graphical abstract

**Figure:**
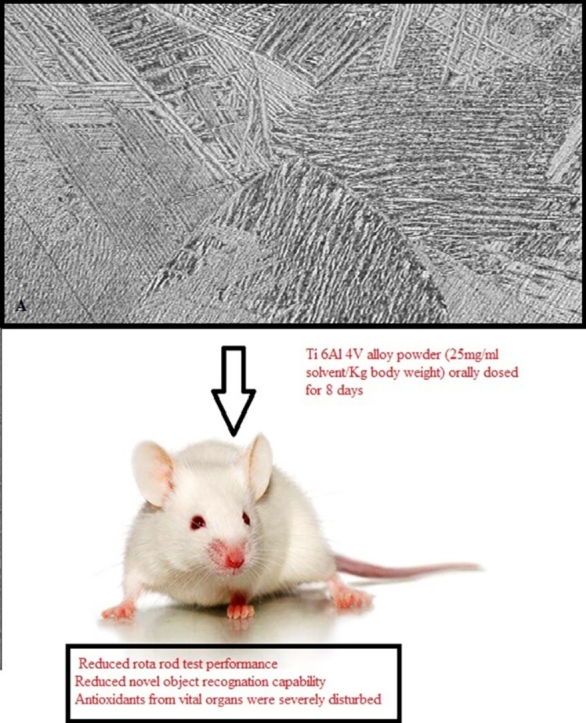

## 1 Introduction

In surgical implants, metals and alloys are the most common materials [1]. Titanium alloys have good mechanical properties and they are considered to be biocompatibile. Hence, they are the materials of choice for the production of implant materials. Titanium alloys have low densities imparting them high specific strength-to-weight ratios that makes them lighter and stronger and even in some cases, they have cryogenic properties [2,3]. They are widely used in aerospace and marine applications, medicines and in production of sports equipment [4,5]. Titanium based implants are very commonly used in dentistry and orthopedic treatments, for spinal and maxillofacial reconstructions, joint arthroplasties and dental prostheses [6,7].

Metallic implants are susceptible to corrosion when they are exposed to the electrolytic environment in human body [8]. With the passage of time, freely corroding implant materials are replaced with corrosion resistant super alloys but still deleterious corrosion processes have been reported in certain clinical cases [9]. In theory, a metallic implant should be completely inert in the living system but unfortunately that happens least frequently and increased concentrations are metals are detected in system that are associated with the implanted materials causing abnormal biological processes in host system [10,11]. The process of corrosion starts as soon as implanted material comes in contact with the extracellular fluids [12]. The human body is a hostile environment containing water, complex organic compounds, lymph, saliva, plasma, and a variety of ions and can cause corrosion by electro chemical reactions with implants [13,14]. It has been reported that release of vanadium ions from alloys by the passive dissolution can cause discoloration of the surrounding tissue or they may elicit an inflammatory response that causes pain and may even lead to loosening owing to osteolysis [15]. Toxicity of vanadium in Ti‐6Al-4V alloy is always a serious concern. Thompson and Puleo [16] had documented that sub lethal doses of the ionic constituents of Ti-6Al-4V alloy can effect the expression of the osteoblastes and deposition of a mineralized matrix. They have concluded that under *in vitro* conditions, ions associated with Ti-6Al-4V alloy can inhibit the normal differentiation of stromal cells in bone marrow into mature osteoblasts. It has been proposed that ions released from implants in vivo may leade to implant failure by hindering the normal bone deposition process. Recently Challa et al. [17] has studied biological properties of Ti‐6Al‐4V alloy and have compared them with Ti‐6 A l‐7Nb alloy and reported that Ti‐6Al‐7Nb was characterized by superior cell attachment, viability, proliferation, morphology and spread than Ti‐6Al‐4V alloy. Based on their results they have suggested that next generation of titanium alloys must be niobium‐containing alloy. The physical and chemical properties of Ti-6Al-4V alloy has been extensively documented but limited information is available regarding their effects on behavior, blood chemistry and antioxidant profile in vital organs of model organism like albino mice. These interesting findings about this commonly used alloy has led us to conducted this study to evaluate the biocompatibility of Ti 6Al 4V alloy powder in mice.

## 2 Materials and methdology

### 2.1 Synthesis of alloy powder

Two samples of 10 g each of Titanium based alloy containing 6% aluminum and 4% vanadium by weight (Ti-6Al-4V) was prepared by melting in an electric arc furnace. All metals were of >99.9% purity and were from sigma Aldrich. The metals were weighed to a precision of 0.0001 g before mixing. Prior to melting the furnace chamber was evacuated to 10^−5^ mbar vacuum and then filled with argon gas. To achieve homogeneity, melting was repeated four times after over turning the button. After melting samples were weighed and weight loss was found to be negligible. The samples were then heated to 1100 °C in vacuum in a Nabertherm tube furnace for 8 h for homogenization. Furnace cooled samples were then prepared for biocompatibility test as well as other characterization tests. About 1 g powder was prepared for x ray diffraction and biocompatibility testing from one the button while the other button was prepared for optical microscopy as well as scanning electron microscopy through multi stage polishing with final polishing carried out with 50 μm diamond paste. The sample was then etched with freshly prepared Kroll’s reagent (1.5 ml HF, 4 ml HNO_3_, 94 ml H_2_O) for 30 s. Optical microscopy was done on LECO LX31 inverted microscope and scanning electron microscopy along with EDAX was performed on Hitachi S-300 SEM.

### 2.2 Experimental animals

Male and female albino mice (BALB/c strain; 7 weeks old) were used as experimental subjects and provided with standard rodent diet and water *ad libitum*. The room temperature was maintained at 22 ± 1 °C. The light dark rhythm was maintained at 14:10 h. All the experimental protocols and animal handling procedures were approved by the ethical committee of Institute of Pure and Applied Biology at Bahauddin Zakariya University Multan (Pakistan).

### 2.3 Experimental design

Mice were divided into two groups (N = 12 for each group). Control group orally received saline solution (0.9% NaCl) [N = 12, male and female 6 each] while the experimental group was orally treated with 25 mg/ml solvent/Kg body weight of Ti-6 A l-4 V alloy powder for eight days through gavage tube [N = 12, male and female 6 each]. A series of neurological (open field, rota rod, novel object and light-dark box) tests were conducted in both groups followed by hematobiochemical testing.

### 2.4 Rota rod

Rota Rod test was performed by using a locally manufactured apparatus that contained a rotating drum with constant acceleration of 40 rpm. Three tainting trials were provided to each mouse followed by three experimental trials. Mean time spent on rotating drum was compared between the control and Ti 6 A l 4 V alloy powder treated groups following Allahyar et al. [18].

### 2.5 Open field test

Open field test is commonly used to assess exploratory behavior and anxiety in rodents [14]. A computational tracking system, Any-Maze (Stoeling, USA) connected with video camera (XPod-058, China) was employed to detect the behavior of mice in the open field chamber (locally manufactured; 40 cm × 40 cm × 70 cm) for 10 min. Means speed (m/s), Mobile episodes, time mobile and time immobile (seconds), Rotations: Clockwise and anticlockwise were recorded following Iqbal et al. [19]

### 2.6 Novel object recognition test

During open field test, two objects were placed in the opposite corners of locally manufactured field (40 × 40 cm with 70 cm high walls). Each mouse was examined twice. In the first trial, mouse was placed in the middle of chamber and was allowed to explore the area for five minutes. Line cross, approach to object A, approach to object B, time spent near object A, time spent near object B and stretch attend frequency were recorded. In the second trial, the procedure was repeated replacing one of the objects with novel one and same paremeters for recorded again following Zhang et al. [20].

### 2.7 Light and dark Box test

The locally manufactured light/dark box test equipment (45 × 27 × 27 cm) was used to study exploratory behavior and it was consisted of a small dark chamber (18 × 27 cm) and large light chamber (27 × 27 cm). The two chambers were connected by an opening (7.5 × 7.5 cm) located in the center of the dividing wall. Mouse was placed in the center of light chamber keeping its snout facing the opening in the wall. Time spent in each chamber, rearing, stretch attend reflex, transition frequency, defecation and urination were recorded over a five minutes’ test following Zahra et al. [21]

### 2.8 Blood and serum collection and analysis from mice

Following the behavioral testing, animals were sacrificed under 3% isoflurane inhalation and blood samples were collected from retro-orbital sinus. Blood was divided into two parts; part one was used for the determination of complete blood count by using SYSMEX auto analyzer (Japan). While second part was used for serum isolationHDL cholesterol, LDL cholesterol, creatinine, cholesterol and triglycerides were determined by using Beckman coulter chemistry analyzer (CX pro., USA) for both treatments following Tabish et al. [22].

### 2.9 Antioxidants determination in vital organs

Following sacrifice, kidney, liver, brain, heart and lungs were surgically isolated from each mouse, rinsed in saline solution and immediately stored at −20 °C until the determination of antioxidant parameters [superoxide dismutase (SOD), lipid peroxidation and Catalase (CAT)] following Chidambara et al. [23] and Haider et al. [24].

### 2.10 Data analysis

Minitab (Version17) was used for the analysis of results. All the data are presented as mean ± standard deviation (SD). Significance level was set at p < 0.05. Student *t*-test was calculated to compare studied parameters of behavior, antioxidants, complete blood count and serum between Ti 6Al 4V alloy powder treatment and the untreated control group.

## 3 Results

### 3.1 Confirmation of titanium, aluminum and vanadium (Ti 6Al 4V) alloy

**Fig. 1:**
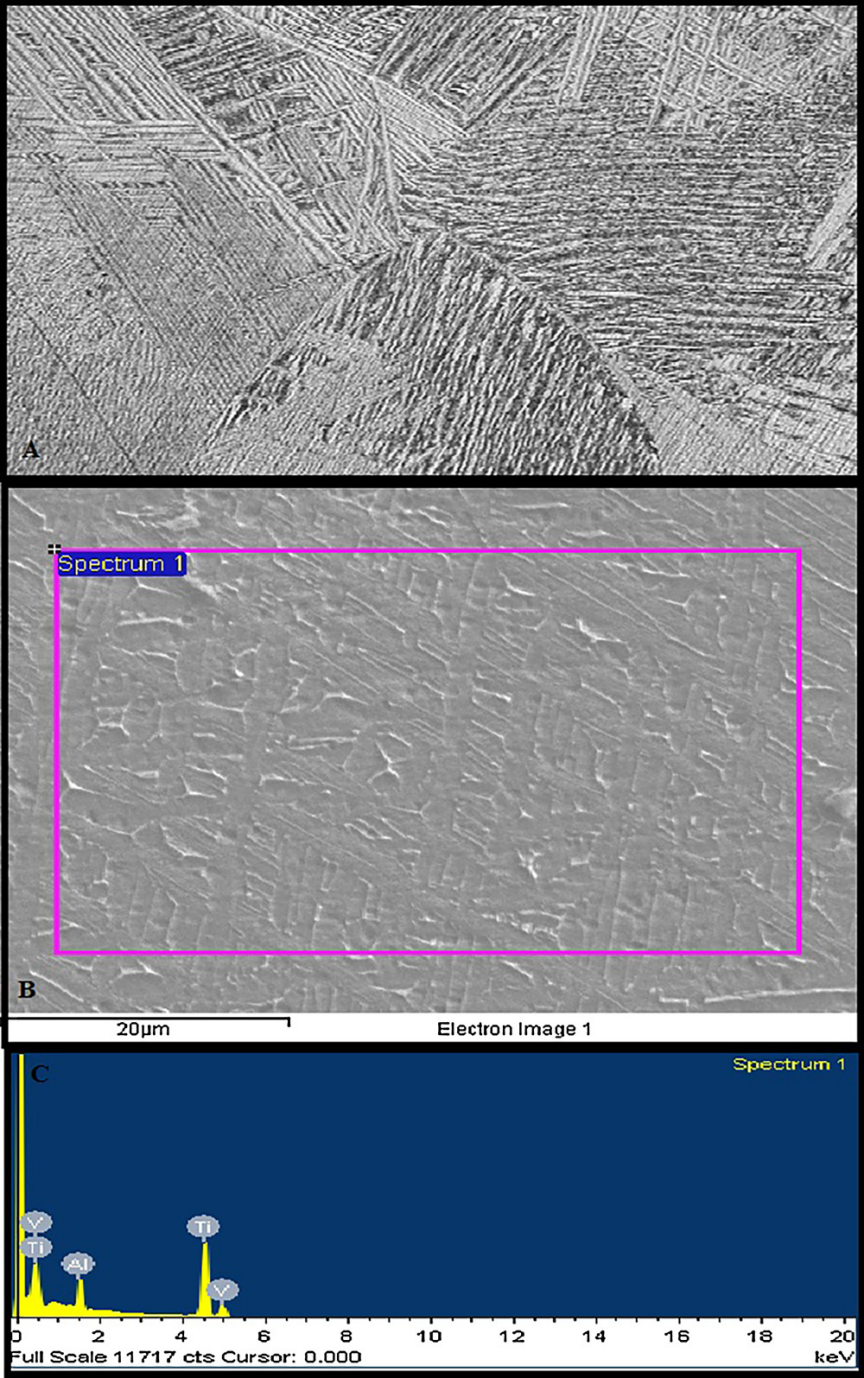
(A) Basket-weave" or Widmanstätten microstructure of homogenized Ti-6Al-4V alloy (100 x). (B) Scanning electron micrograph along with (C) EDAX scan showing that the alloys were composed of selected constituents only.

Optical micrograph revealed the typical “Basket weave like” also called Widmanstätten microstructure (Fig. 1A). The microstructure was composed of alpha phase lamellae dispersed in large beta phase grains. SEM micrograph (Fig. 1B) and the EDAX result (Fig. 1C) clearly indicated that the material was free from all contaminations.

### 3.2 Rota rod test

**Fig. 2:**
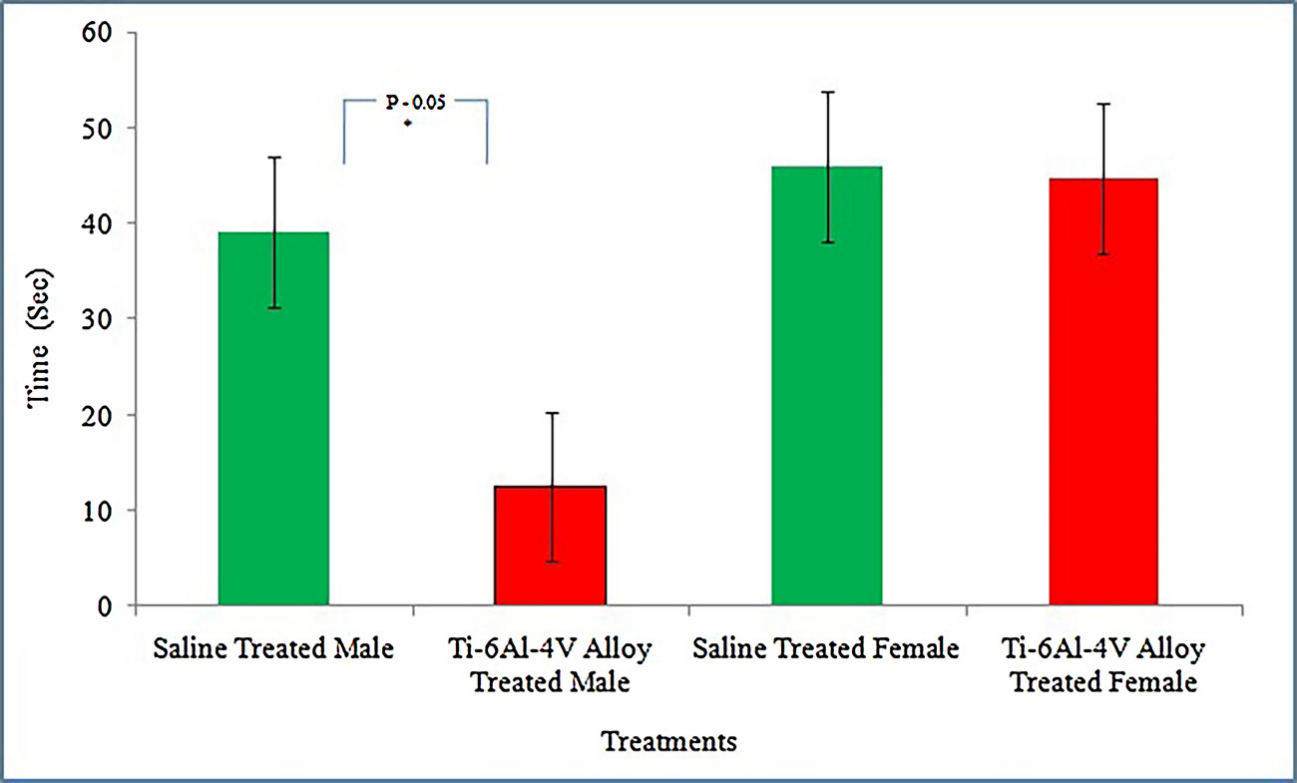
Comparison of Rota rod test performance between 25 mg/ml solvent/Kg body weight of Ti-6Al-4V alloy powder and saline treated albino mice of both genders. N = 6 for each treatment. Data is expressed as mean ± standard deviation. P- value represents the results for two sample *t*-test calculated for each parameter.

Analysis of results revealed that rota rod test performance was significantly decreased (P = 0.05) in male mice treated with25 mg/ml solvent/Kg body weight of Ti-6 A l-4 V alloy powder for eight days as compared to control group. While performance of this test remained unaffected (P > 0.05) when compared between alloy powder treated and untreated female albino mice (Fig. 2).

### 3.3 Open field test

Data analysis revealed that open field test parameters varied non-significantly (P > 0.05) when compared between Ti-6 A l-4 V alloy powder treated mice of both genders with their respective control group (Supplementary Table S1).

### 3.4 Novel object recognition test

Analysis of novel object recognition test results revealed that the studied parameters varied non-significantly (P > 0.05) when compared between alloy powder treated male mice with their control group during both trials (Supplementary Table S2A and B). In case of female, it was mice observed that mice treated with Ti-6 A l-4 V alloy powder had approached novel object during trail two significantly less frequently (P = 0.04) than their control group (Supplementary Table S2B).

### 3.5 Light and dark Box test

Data analysis indicated that all studied parameters of light dark test varied non- significantly (P > 0.05) when compared between Ti-6 A l-4 V alloy powder treated mice of both genders with their respective control group (Supplementary Table S3).

### 3.6 Complete blood count analysis

Data analysis revealed that all the studied parameters of hematological profile varied non- significantly (P > 0.05) when compared between Ti-6 A l-4 V alloy powder treated and untreated albino mice (Supplementary Table S4).

### 3.7 Serum biochemical profile analysis

All the studied parameters varied non-significantly (P > 0.05) when compared between Ti 6 A l 4 V alloy powder treated and untreated albino mice of both genders (Supplementary Table S5).

### 3.8 Antioxidant analysis

Data analysis revealed that concentration of SOD in liver (P = 0.008), heart (P = 0.01) and lungs (P = 0.05) was significantly elevated while catalase concentration in liver (P = 0.001) was significantly decreased in female albino mice that were exposed to 25 mg/ml solvent/kg body weight of Ti 6 A l 4 V alloy powder as compared to control group (Table 1). In case of male albino mice, SOD concentration in lungs was only parameters that significantly reduced (P = 0.05) in mice exposed to Ti 6 A l 4 V alloy powder as compared to control group. All other studied metabolites varied non-significantly (P > 0.05) between the two treatments in all the analyzed organs of both genders (Table 1).

**Table 1:** Comparison of various studied antioxidant parameters between 25mg/ml solvent/Kg body weight of Ti-6Al-4V alloy powder and saline treated albino mice of both genders. (N = 6 for each treatment). All values are expressed as mean ± standard deviation. P-value presents the results of 2 sample *t*-test conducted for each parameter between the two treated groups.

Gender	Metabolites	Brain			Heart			Liver			Kidney			Lungs		
		Saline treated Control	Ti-6Al-4Nb Treated	P value	Saline treated Control	Ti-6Al-4Nb Treated	P value	Saline treated Control	Ti-6Al-4Nb Treated	P value	Saline treated Control	Ti-6Al-4Nb Treated	P value	Saline treated Control	Ti-6Al-4Nb Treated	P value
Female Mice	Superoxide dismutase (unit/gm)	8.43 ± 0.86	10.73 ± 1	0.1	0.53 ± 0.92	2.37 ± 0.42	0.008**	0.76 ± 0.09	1.7 ± 0.26	0.01**	0.972± 0.36	1.659 ± 0.29	0.2	0.758 ± 0.15	1.203 ± 0.14	0.05*
	Malonaldehyde (picomol/gm)	72.0± 7.1	76.6± 6.9	0.7	60.3 ± 18	54.3 ± 12	0.8	36.3 ± 7.0	34.7 ± 5.4	0.9	47.3 ± 7.7	44.3 ± 4.9	0.8	38.90 ± 2.6	44.07 ± 3.9	0.3
	Catalase (mg/dL)	31.90± 1.1	34.38 ± 1.8	0.3	30.8± 1.1	29.3 ± 1.0	0.3	12.8 ± 0.47	5.81 ± 1.2	0.001**	32.79 ± 0.89	34.85 ± 2.3	0.4	31.63 ± 1.5	32.40± 1.7	0.8
Male Mice	Superoxide dismutase (unit/gm)	6.55 ± 0.58	5.760 ± 0.40	0.3	0.468 ± 0.12	0.463 ± 0.11	1	1.099 ± 0.26	1.271 ± 0.19	0.6	0.460 ± 0.061	0.584 ± 0.057	0.2	2.461 ± 0.20	1.422 ± 0.39	0.05*
	Malonaldehyde (picomol/gm)	71.2 ± 9.8	97.5± 9.6	0.09	56.9 ± 13	74.9 ± 11	0.3	35.1 ± 4.4	39.7± 8.7	0.7	54.3 ± 7.9	47.4 ± 11	0.6	38.8 ± 13	43.6 ± 7.8	0.8
	Catalase (mg/dL)	34.70± 1.5	30.76± 2.0	0.2	29.53 ± 1.6	26.59 ± 1.4	0.2	18.1 ± 5.5	12.06 ± 3.4	0.4	23.00 ± 3.9	18.74± 4.2	0.5	30.47 ± 0.36	30.93 ± 1.7	0.8

P > 0.05 = Non significant, P < 0.05 = least significant (*), P < 0.01 = significant (**).

### 3.9 Body weight analysis

Analysis of data revealed that body weight varied non- significantly (p > 0.05) when compared between Ti-6Al-4V alloy powder treated and untreated albino mice of both genders during the whole experiment (Supplementary Fig. S1).

## 4 Discussion

Due to the presence of large number of ions and proteins in the bosy, usually the surgical implants under goes corrosion when comes in contact with them. These interactions leads to oxidation of metallic alloy components to their ionic forms and dissolved oxygen is reduced to hydroxide ions [25]. There are many factors that regulate the amount of metal ions released from implants during corrosion including the environmental conditions around implant, corrosion resistance of the metal, mechanical factors (i.e., pre-existing cracks, surface abrasion and film adhesion), electrochemical effects (i.e., galvanic effects, pitting, applied potential, or crevices) and the dense cell concentrations around implants [26]. Thisfindings have led us to explore the biocompatibility of this alloy further and present study was conducted to report the effect of 25 mg/ml solvent/Kg body weight of Ti-6Al-4V alloy powder following supplementation for 8 days on the behavior and biochemistry of albino mice.

Analysis of results indicated drastic effect of alloy powder supplementation on performance of two neurological tests in a gender specific manner. It was observed that neuromuscular coordination of male mice during Rota rod test (Fig. 2) and novel object recognition capability of female albino mice (Supplementary Table S2) was compromised after supplementation of 25 mg/ml solvent/Kg body weight of Ti-6Al-4V alloy powder for 8 days indicating that the alloy powder was capable of crossing the blood brain barrier and was capable to affect the brain functioning.

Hematological parameters are affected by environmental factors, stress, and nutritional deficiencies and are generally used as good health indicator and are used for the diagnosis of various diseases [27]. Serum plays an important role in the regulation of body temperature, pH and transport of small molecules in the body and hence serves as good indicator of health [28]. All the studied hematological parameters remained unaffected during present study when compared between Ti-6Al-4V alloy powder treated and untreated albino mice of both genders (Supplementary Tables S4, S5) indicating that probably exposure time was not long enough to disturb the blood chemistry of albino mice.

Reactive Oxygen Species (ROS) are by products of metabolism and include hydroxyl radicals (OH), hydrogen peroxide (H_2_O_2_), superoxide anion (O2−) and nitric oxide (NO). They have high chemical reactivity that leads to lipid peroxidation and oxidation of some enzymes and massive protein oxidation and degradation [29]. Antioxidant defenses in the cell are to neutralize the negative effects of free radicals [30,31]. Defenses against ROS-induced damage include the enzymes catalase and glutathione peroxidase [both of which remove H_2_O_2_, as well as Superoxide dismutase (SOD), which catalyzes the dismutation of O_2_•^−^ to form H_2_O_2_ and O_2_•^−^] [32]. It has been reported that cells in peri-implant tissue as well as metal corrosion can induce reactive oxygen species (ROS) formation, thus contributing to an oxidative microenvironment around an implant that can lead to variety of pathological conditions [33]. These findings led us to study the effect of Ti-6Al-4V alloy on the antioxidant profile in five vital organs of albino mice.

Analysis of our results indicated that concentration of SOD in liver, heart and lungs was significantly elevated while catalase concentration in liver was significantly decreased in female albino mice that were exposed to 25 mg/ml solvent/kg body weight of Ti 6Al 4V alloy powder (Table 1). In case of male albino mice, SOD concentration in lungs was only parameters that significantly reduced in mice exposed to Ti 6Al 4V alloy powder. This increase in SOD activity reflects the change in oxidative metabolism and increased H₂O₂ production in cell.

## 5 Conclusion

In conclusion, our results indicated that 25 mg/ml solvent/Kg body weight of Ti-6Al-4V alloy powder supplementation had adversely affected rota rod and novel object recognition test performance in male and female albino mice respectively. Analysis of antioxidant metabolites in vital organs has indicated that the applied dose of Ti-6Al-4V alloy powder has disturbed the H₂O₂ and lipid peroxidation associated metabolic pathways in albino mice, especially in female mice. As this alloy is part of several surgical implants, so it is recommended that their effects should be explored in further details under variable dose and exposure time conditions to get a broader vision regarding their biocompatibility in living systems.

## Conflict of interest

Authors declare that they do not have conflict of interest of any sort with anyone.

## Transparency document

Transparency document
