# Supplementary material for: Short term exposure to titanium, aluminum and vanadium (Ti 6Al 4V) alloy powder drastically affects behavior and antioxidant metabolites in vital organs of male albino mice
Source: Toxicol Rep. 2018 Jun 13;5:765–70. doi: 10.1016/j.toxrep.2018.06.006 (PMC6031284; doi:10.1016/j.toxrep.2018.06.006)

**Short term exposure to Titanium, Aluminum and Vanadium** (**Ti 6Al 4V) alloy powder drastically affects behavior and antioxidant metabolites in vital organs of male albino mice**

Ghulam Khadija ^1, #^, Ayisha Saleem ^2, #^, Zafrin Akhtar ^1,^ Zahra Naqvi ^1^, Maham Gull ^1^, Mahnoor Masood ^1^, Sana Mukhtar ^1^, Momna Batool ^1^, Nida Saleem ^1^, Tahir Rasheed ^1^, Naira Nizam ^1^, Ather Ibrahim ^1,^ *, Furhan Iqbal ^2,^ *

1 *Institute of Pure and Applied Biology, Zoology Division. Bahauddin Zakariya University, Multan, Pakistan*

2 *Institute of Advanced Materials. Bahauddin Zakariya University, Multan, Pakistan*

**Supplementary Table 1** Comparison of various studied parameters of open field test between 25mg/ml solvent/Kg body weight of Ti-6Al-4V alloy powder and saline treated albino mice of both genders. N = 6 for each treatment. Data is expressed as mean ± standard deviation. P- value represents the results for two sample t – test calculated for each parameter.

| **Studied parameters** | **Female mice** | | | **Male mice** | | |
| --- | --- | --- | --- | --- | --- | --- |
|  | **Saline treated** | **Ti-6Al-4V Alloy treatment** | **P-value** | **Saline treated** | **Ti-6Al-4V Alloy treatment** | **P-value** |
| **Distance (m)** | _27.73 ± 1.5_ | _25.85 ±1.7_ | _0.4_ | _25.17 ± 8.20_ | _28.9 ± 12.9_ | _0.6_ |
| **Mean Speed (m / s)** | _0.046 ±0.0027_ | _0.043 ±0.0027_ | _0.4_ | _0.04 ± 0.01_ | _0.05 ± 0.02_ | _0.6_ |
| **Time mobile (sec)** | _504.1 ± 21_ | _474.8 ±29_ | _0.4_ | _474.7 ± 67.3_ | _488.5 ± 82.0_ | 0.8 |
| **Time immobile (sec)** | _95.9± 21_ | _125.2±29_ | _0.4_ | _125.4 ± 67.3_ | _111.5 ± 82_ | 0.8 |
| **Mobile episodes** | _21.50 ± 2.2_ | _23.33 ± 3.0_ | _0.6_ | _26.8 ± 11.1_ | _22.7 ± 10.6_ | 0.5 |
| **Immobile episodes** | _20.67 ± 2.2_ | _22.67 ± 3.0_ | _0.6_ | _26.0 ± 11.4_ | _21.7 ± 10.6_ | 0.5 |
| **Rotations** | _33.67 ± 1.8_ | _27.33 ± 2.5_ | _0.07_ | _29.50 ± 6.53_ | _32.5 ± 11.2_ | 0.6 |
| **Clockwise rotations** | _17.33 ± 1.9_ | _14.83 ± 1.4_ | _0.3_ | _14.0 ± 3.35_ | _15.3 ± 8.2_ | 0.7 |
| **Anticlockwise rotation** | _16.33 ± 1.5_ | _12.50 ± 1.9_ | _0.2_ | _15.5 ± 3.51_ | _17.17 ± 7.6_ | 0.6 |
|  |  |  |  |  |  |  |

P > 0.05 = Non significant

**Supplementary Table 2A** Comparison of various studied parameters during first trial of novel object test between 25mg/ml solvent/Kg body weight of Ti-6Al-4V alloy powder and saline treated albino mice of both genders. N = 6 for each treatment. Data is expressed as mean ± standard deviation. P- value represents the results for two sample t – test calculated for each parameter.

| **Studied parameters** | **Female mice** | | | **Male mice** | | |
| --- | --- | --- | --- | --- | --- | --- |
|  | **Saline treated** | **Ti-6Al-4V Alloy treatment** |  | **Saline treated** | **Ti-6Al-4V Alloy treatment** |  |
| **Line cross** | 26.83 ±6.40 | 27.0 ±3.35 | 1 | 22.3 ± 10.8 | 20.0 ± 6.03 | 0.7 |
| **Stretch attend reflex** | 38.3± 11.7 | 42.5 ± 13.3 | 0.6 | 37.8 ± 18.9 | 31.5 ± 10.3 | 0.5 |
| **Approaches object A** | 15.00 ± 3.85 | 12.8 ± 2.04 | 0.3 | 11.0 ± 5.22 | 10.0 ± 2.61 | 0.7 |
| **Approaches object B** | 12.33 ± 6.09 | 11.83±4.62 | 0.9 | 9.17 ± 4.54 | 12.67 ± 4.76 | 0.2 |
| **Time object A (sec)** | 67.2± 14.6 | 53.8 ± 11.8 | 0.1 | 47.7 ± 18.2 | 47.0 ± 14.8 | 1 |
| **Time object B (sec)** | 46.2±18.7 | 54.7 ± 20.6 | 0.5 | 40.2 ± 23.3 | 66.2 ± 26.4 | 0.1 |

P > 0.05 = Non significant

**Supplementary Table 2B** Comparison of various studied parameters during second trial of novel object test between 25mg/ml solvent/Kg body weight of Ti-6Al-4V alloy powder and saline treated albino mice of both genders. N = 6 for each treatment. Data is expressed as mean ± standard deviation. P- value represents the results for two sample t – test calculated for each parameter.

| **Studied parameters** | **Female mice** | | | **Male mice** | | |
| --- | --- | --- | --- | --- | --- | --- |
|  | **Saline treated** | **Ti-6Al-4V Alloy treatment** |  | **Saline treated** | **Ti-6Al-4V Alloy treatment** |  |
| **Line cross** | 14.83 ± 6.18 | 12.00 ± 2.83 | 0.3 | 19.67 ± 9.35 | 15.67 ± 4.80 | 0.4 |
| **Stretch attend reflex** | 28.0 ±14.8 | 29.3± 18.1 | 0.9 | 46.0 ± 24.6 | 34.50 ± 9.33 | 0.3 |
| **Approaches object A** | 7.33± 2.50 | 5.83± 3.31 | 0.4 | 6.83 ± 4.45 | 7.50 ± 2.35 | 0.8 |
| **Approaches Novel object** | 8.17 ±2.93 | 4.67 ± 1.75 | 0.04 * | 9.33 ± 3.50 | 8.67 ± 4.37 | 0.8 |
| **Time Old object (sec)** | 42.3 ± 24.8 | 32.7 ± 23.3 | 0.5 | 43.0 ± 30.9 | 61.8 ± 36.2 | 0.4 |
| **Time Novel object (sec)** | 33.3 ±15.1 | 75.3 ± 81.6 | 0.3 | 56.3 ± 18.0 | 70.7 ± 17.7 | 0.2 |

P > 0.05 = Non significant**;** P < 0.01 = Least significant (*); P < 0.01 = Significant (**)

**Supplementary Table 3** Comparison of various studied parameters of light dark box test between 25mg/ml solvent/Kg body weight of Ti-6Al-4V alloy powder and saline treated albino mice of both genders. N = 6 for each treatment. Data is expressed as mean ± standard deviation. P- value represents the results for two sample t – test calculated for each parameter.

| **Studied parameters** | **Female mice** | | | **Male mice** | | |
| --- | --- | --- | --- | --- | --- | --- |
|  | **Saline treated** | **Ti-6Al-4V Alloy treatment** | **P-value** | **Saline treated** | **Ti-6Al-4V Alloy treatment** | **P-value** |
| **Transition frequency** | 13.83 ±2.2 | 13.50 ± 1.8 | 0.9 | 15.83 ± 4.79 | 20.67 ± 8.02 | 0.2 |
| **Rearing frequency** | 15.3 ± 11 | 15.5 ± 8.7 | 1 | 5.17 ± 2.56 | 2.83 ± 2.93 | 0.2 |
| **Stretch attend frequency** | 54.7 ±8.7 | 57.3 ± 11 | 0.9 | 46.2 ± 20.8 | 40.5 ± 21.1 | 0.7 |
| **Time in dark (sec)** | 154 ±15 | 173 ±17 | 0.4 | 180.8 ± 48.5 | 199.7 ± 62.7 | 0.6 |
| **Time in light (sec)** | 144.3 ± 15 | 125.3 ±17 | 0.4 | 119.2 ± 48.5 | 100.3 ± 62.7 | 0.6 |
| **Urination** | 0 ± 0 | 0 ± 0 | 0 | 1.33 ± 1.51 | 0.50 ± 1.22 | 0.3 |
| **Defection** | 1.00 ± 0.63 | 1.67 ±1.7 | 0.7 | 5.17 ± 5.46 | 0.33 ± 0.82 | 0.08 |

P > 0.05 = Non significant

**Supplementary Table 4** Comparison of various studied parameters of complete blood count between 25mg/ml solvent/Kg body weight of Ti-6Al-4V alloy powder and saline treated albino mice of both genders. N = 6 for each treatment. Data is expressed as mean ± standard deviation. P- value represents the results for two sample t – test calculated for each parameter

| **Studied parameters** | **Female mice** | | | | | | **Male mice** | | | | |  |
| --- | --- | --- | --- | --- | --- | --- | --- | --- | --- | --- | --- | --- |
|  | **Saline treated** | | **Ti-6Al-4V Alloy treatment** | | **P value** | | **Saline treated** | **Ti-6Al-4V Alloy treatment** | | | **P value** |  |
| **RBC (x 10^6^ µL^-1^)** | | 6.51±0.53 | | 7.02±0.5 | | 0.5 | 6.55 ± 1.1 | | 6.18 ± 1.5 | 0.8 | | |
| **WBC (x 10^3^ µL^-1^)** | | 7.07±1.7 | | 10.8±3.3 | | 0.4 | 8.18 ± 0.27 | | 8.87 ± 3.4 | 0.2 | | |
| **Hemoglobin (gdL^-1^)** | | 12.03±1.1 | | 12.97±1.1 | | 0.6 | 11.52 ± 1.8 | | 10.87 ± 2.4 | 0.8 | | |
| **MCV (fl)** | | 53.33±3.4 | | 54.58±3.1 | | 0.8 | 58.78 ± 2.8 | | 61.68± 2.9 | 0.5 | | |
| **MCH (pg)** | | 18.60±1.1 | | 18.57±1 | | 1.0 | 17.83± 2.0 | | 18.60 ± 0.95 | 0.5 | | |
| **MCHC (gdL^-1^)** | | 36.3±4.6 | | 34.93±3.6 | | 0.8 | 30.78 ± 2.0 | | 30.47 ± 2.1 | 0.9 | | |
| **Platelets (x 10^3^ µL^-1^)** | | 327±73 | | 532±159 | | 0.3 | 448± 131 | | 534 ± 136 | 0.6 | | |
| **Lymphocytes (x10^3^ µL^-1^)** | | 5.85±1.3 | | 8.45±2.2 | | 0.3 | 2.783 ± 0.2 | | 6.87 ± 2.5 | 0.2 | | |
| **PCT (%)** | | 0.233±0.045 | | 0.386±0.11 | | 0.2 | 0.332±0.096 | | 0.390±0.099 | 0.7 | | |
| **HCT (%)** | | 34.82±3.5 | | 38.75±4.0 | | 0.5 | 38.2±6.6 | | 38.1±9.5 | 1.0 | | |
| **Monocytes (x10^3^ µL^-1^)** | | 1.067±0.18 | | 1.050±0.30 | | 1.0 | 0.0167±0.017 | | 0.133±0.056 | 0.1 | | |
| **Granulocytes (x10^3^ µL^-1^)** | | 1.15±0.42 | | 2.17±1.1 | | 0.4 | 87.5±2.6 | | 80.85±3.3 | 0.1 | | |
| **Granulocytes (%)** | | 14.00±2.6 | | 14.1±4.3 | | 1.0 | 11.60 ± 2.4 | | 17.77± 8.08 | 0.2 | | |
| **Lymphocytes (%)** | | 84.93±2.7 | | 84.8±4.6 | | 1.0 | 87.5± 2.6 | | 80.85 ± 3.3 | 0.1 | | |
| **Monocytes (%)** | | 1.067±0.18 | | 1.050±0.30 | | 1.0 | 0.9± 0.16 | | 1.383 ± 0.21 | 0.1 | | |
| **RDW-SD (µm^3)^** | | 40.77±3.2 | | 40.45±3.0 | | 0.9 | 43.7± 4.5 | | 51.73 ± 3.7 | 0.2 | | |
| **RDW (%)** | | 21.28±1.3 | | 21.07±1.7 | | 0.9 | 20.15 ± 1.3 | | 22.28 ± 1.4 | 0.3 | | |
| **PDW (%)** | | 18.32±1.9 | | 20.42±3.5 | | 0.6 | 18.18 ± 2.6 | | 16.63± 2.8 | 0.7 | | |
| **MPV (fl)** | | 7.333±0.22 | | 7.58±0.46 | | 0.6 | 7.367± 0.20 | | 7.150 ± 0.32 | 0.6 | | |

RBC: red blood cell, WBC: white blood cell, HCT: hematocrit, MCV: mean corpuscular volume, MCH: mean corpuscular hemoglobin, MCHC: mean corpuscular hemoglobin concentration, RDW: Red cell distribution width, PDW: Platelet distribution width, MPV: Mean platelet volume, PCT: Platelet Crit.

P > 0.05 = Non-significant.

**Supplementary Table 5** Comparison of various studied parameters of serum biochemical profile between 25mg/ml solvent/Kg body weight of Ti-6Al-4V alloy powder and saline treated albino mice of both genders. N = 6 for each treatment. Data is expressed as mean ± standard deviation. P- value represents the results for two sample t – test calculated for each parameter

| **Studied parameters** | **Male mice** | | | | | | **Female mice** | | | | | | | | | |
| --- | --- | --- | --- | --- | --- | --- | --- | --- | --- | --- | --- | --- | --- | --- | --- | --- |
|  | **Saline treatment** | | **Ti-6Al-4V Alloy treatment** | | **P value** | | **Saline treatment** | | **Ti-6Al-4V Alloy treatment** | | | **P value** | | | | |
| **HDL Cholesterol(mg/dl)** | | 42.85 ± 1.9 | | 41.92± 5.3 | | 0.9 | | 69.7± 30 | | | 41.31± 1.6 | | | 0.4 | |  |
| **LDL Cholesterol(g/dl)** | 113.49 ± 2.6 | | 69.86± 5.9 | | 0.09 | | 101.2± 23 | | | 86.30± 6.6 | | | 0.6 | |  |  |
| **Triglyceride(mg/dl)** | 64.68 ± 4.3 | | 101.2 ± 27 | | 0.3 | | 133.0 ± 12 | | | 153.4 ± 24 | | | 0.5 | |  |  |
| **Creatinine (mg/dl)** | 0.348±0.097 | | 0.39 ± 0.12 | | 0. 8 | | 0.315±0.065 | | | 0.374 ± 0.21 | | | 0.8 | |  |  |
| **Cholesterol (mg/dl)** | 217.7 ± 22 | | 213.9 ± 33 | | 0.9 | | 165.6± 14 | | | 164.4± 5.1 | | | 0.9 | |  |  |

P > 0.05 = Non significant

**Supplementary Figure 1** Comparison of body weight between 25mg/ml solvent/Kg body weight Ti-6Al-4V alloy powder and saline albino mice of both genders during experiment. N = 6 for each treatment. Data is expressed as mean ± standard deviation. P values represent the results of 2 sample t-tests calculated for each gender at specific time point.


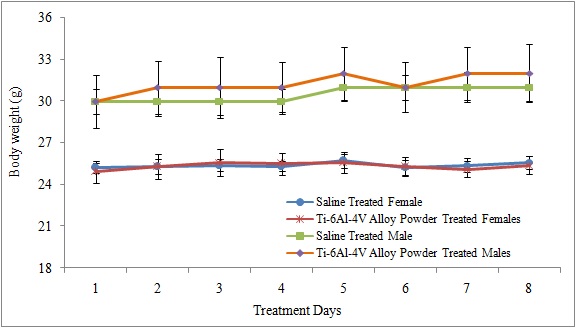

Supplement: Supplementary file 2 [file mmc2.docx]
